# Clinical oral microbiology and antimicrobial susceptibility – does it matter?

**DOI:** 10.1080/20002297.2017.1325228

**Published:** 2017-06-01

**Authors:** Andrew Smith

**Affiliations:** ^a^ College of Medical, Veterinary & Life Sciences, University of Glasgow, UK

## Abstract

Little surveillance data is undertaken on the antimicrobial susceptibility of isolates from acute dento-alveolar infections. We present data collated from routine diagnostic microbiology specimens submitted over a 27 year period in Glasgow, UK. Over the study period isolates were identified using the API system, VITEK 2 and MALDI-TOF. Antimicrobial susceptibility tests were undertaken using combinations of Stokes method and/or E-tests. Baseline data based on Lewis et al., (JAC 23,69-77 1989) and compared to isolates from 1998-2015. From 621 specimens, we isolated Anginosus group streptococci (n = 196), Mitis group Streptococci (n = 448), anaerobic streptococci (n = 116), Fusobacteria (n = 121) and Prevotella (n = 140) species. No discernable increase in resistance patterns over the 27 year time period of analysis was observed. A 6 month audit (May-Oct 2016) in Glasgow recorded 37 patients hospital admissions for 142 bed days (mean 3.5 days) to incise and drain severe odontogenic infections. But, dental practitioners in England (2015) prescribed 10,717 items of cephalosporins and 18, 630 items of augmentin (Source: Health and Social Care Information Centre) – why?

Diagnostic oral microbiology matters in guiding antimicrobial stewardship programs but more collaborative working on processing methods and routine susceptibility data would help overcome limited data from single centers.

